# Hemophagocytic Lymphohistiocytosis Caused by a Severe Epstein-Barr Virus Infection in a Young Patient Presenting With Hiccups

**DOI:** 10.7759/cureus.36199

**Published:** 2023-03-15

**Authors:** Hadi Abou-El-Hassan, Melvin Kantono, Ankur Bhagat, Janie Hu, David Karp, Renard Jerome, Johnny S Randhawa, Drake Shafer, Farbod Farmand

**Affiliations:** 1 Neurology, Arrowhead Regional Medical Center, Colton, USA; 2 Internal Medicine, Arrowhead Regional Medical Center, Colton, USA; 3 Internal Medicine, California University of Science and Medicine, Colton, USA

**Keywords:** immune system dysregulation, cytokine storming, ebv splenomegaly, ebv hlh, hemophagocytic lymphohistiocytosis (hlh)

## Abstract

Hemophagocytic lymphohistiocytosis (HLH) is a life-threatening hyperinflammatory syndrome characterized by a pathologic immune response in the setting of infection, malignancy, acute illness, or any immunological stimulus. Infection is the most common etiology of HLH. HLH involves aberrant activation of lymphocytes and macrophages with resultant hypercytokinemia due to an inappropriately stimulated and ineffective immune response. Here, we present the case of a previously healthy 19-year-old male presenting with hiccups and scleral icterus, who was found to have HLH due to a severe Epstein-Barr virus infection. Despite a morphologically normal bone marrow biopsy, the patient met the diagnostic criteria for HLH, including a low natural killer cell count and elevated soluble interleukin-2 receptor. Notably, ferritin was severely elevated at 85,810 ng/mL. The patient was treated with an induction course of dexamethasone intravenously for eight weeks. Since HLH can progress into multi-organ failure, timely diagnosis and prompt initiation of treatment are critical. Novel disease-modifying therapies and further clinical trials are warranted to treat this potentially fatal immunological disease with multisystem ramifications.

## Introduction

Hemophagocytic lymphohistiocytosis (HLH), initially named histiocytic medullary reticulosis, is a rare, life-threatening disease, characterized by inappropriate activation of the immune system [1]. Natural killer (NK) cells, cytotoxic T-cells, and macrophages are the most dysregulated immune cells in the pathogenesis of HLH causing overexpression of cytokines, widespread inflammation, and multi-organ tissue damage [2]. HLH is often triggered by an infection (49%) [3], malignancy (27%) [4], autoimmune stimulus (7%) [5], or any acute illness [6].

Although HLH commonly affects children with a median age at a diagnosis of 1.8 years [7], it has been reported across all age groups [8]. Adults account for 40% of all cases [9], with a median age at diagnosis of 48 years [10]. HLH presents with a myriad of clinical and laboratory manifestations that include fever, organomegaly, liver injury, consumptive coagulopathy, hypertriglyceridemia, cytopenia, hemophagocytosis, absent NK cell activity, elevations of acute phase reactants, electrolyte abnormalities [11], neurologic dysfunction [12], and cutaneous lesions [13]. Here, we present the case of a previously healthy 19-year-old male presenting with hiccups and scleral icterus who was found to have severe Epstein-Barr virus (EBV)-induced HLH.

## Case presentation

A 19-year-old Hispanic male with no known past medical history presented to the emergency department for a two-week history of hiccups, mid-epigastric abdominal pain, and scleral icterus. Upon examination in the emergency department, blood pressure was 117/73 mmHg, heart rate was 64 beats per minute, the temperature was 36.9 °C (98.4 °F), respiratory rate was 16, oxygen saturation of 99% on room air, blood glucose was 141 mg/dL, and body mass index was 22.1 kg/m^2^. Physical exam was unremarkable except for scleral icterus and mild mid-epigastric tenderness. The patient denied any alcohol consumption, cigarette smoking, or illicit or intravenous drug use. The patient is a senior high school student who lives with his parents. The patient’s family history was non-contributory. EKG showed normal sinus rhythm. Initial laboratory studies were notable for bicytopenia, hypertriglyceridemia, transaminitis, hypofibrinogenemia, and hyperferritinemia (Table 1). The international society on thrombosis and hemostasis score for disseminated intravascular coagulation was less than five. In addition, no schistocytes were noted, the hepatitis viral panel was negative, HIV screening was negative, syphilis IgG/IgM was negative, acetaminophen level was undetectable, and a urine toxicology test was negative.

**Table 1:** Laboratory studies demonstrating the laboratory criteria of hemophagocytic lymphohistiocytosis that our patient met, including bicytopenia, hypertriglyceridemia, hyperferritinemia and high soluble CD25 level. μL: microliter, g: gram, dL: deciliter, fL: femtoliter, mEq: milliequivalent, L: liter, mmol: millimole, mg: milligram, ng: nanogram, mL: milliliter, mIU: milli-international units, pg: picogram, U: units, mcg: microgram, mm: millimeter, hr: hour, BUN: blood urea nitrogen, TSH: thyroid-stimulating hormone, HbA1c: hemoglobin A1C, HDL: high-density lipoprotein, LDL: low-density lipoprotein, INR: international normalized ratio, aPTT: Activated partial thromboplastin time, CD: cluster of differentiation.

Laboratory test	Measured value	Reference range
White blood cells (cells/μL)	700	4,300-11,100
Absolute neutrophil count (cells/μL)	300	> 1,500
Hemoglobin (g/dL)	13.8	12.1-15.1
Hematocrit (%)	42	36-46
Platelet (cells/μL)	92,000	120,000-360,000
Mean corpuscular volume (fL)	88	80-100
Sodium (mEq/L)	137	135-145
Potassium (mEq/L)	4	3.5-5.5
Chloride (mEq/L)	98	98-110
Bicarbonate (mmol/L)	27	24-34
BUN (mg/dL)	9	8-20
Creatinine (mg/dL)	0.61	0.5-1.5
Venous lactate (mmol/L)	0.99	0.5-2.0
Troponin (ng/mL)	< 0.3	0.0-0.30
TSH (mIU/L)	2.39	0.35-5.5
HbA1c (%)	5.8	< 5.7
Vitamin B-12 (pg/mL)	1,877	250-1,100
Folate (ng/mL)	7.7	2.8-25
Cholesterol (mg/dL)	118	< 200
Triglycerides (mg/dL)	528	< 150
HDL (mg/dL)	8	> 40
LDL (mg/dL)	7	< 100
Aspartate aminotransferase (U/L)	514	5-40
Alanine aminotransferase (U/L)	645	5-40
Alkaline phosphatase (U/L)	1,070	35-125
Gamma glutamyl transferase (U/L)	207	5-37
Direct bilirubin (mg/dL)	3.9	0-0.4
Albumin (g/dL)	3.6	3.5-4.9
Total protein (g/dL)	6.9	6-8
INR	1.16	< 1.1
aPTT (seconds)	43.2	25.4-36.8
D-Dimer (mcg/mL)	1,090	0-250
Fibrinogen (mg/dL)	159	174-482
Erythrocyte sedimentation rate (mm/hr)	14	0-10
C-reactive protein (mg/L)	0.4	< 0.5
Haptoglobin (mg/dL)	<8	43-212
Lactate dehydrogenase (U/L)	1,224	120-230
Iron (mcg/dL)	90	50-160
Ferritin (ng/mL)	85,810	20-300
Copper (mcg/dL)	144	70-175
Ceruloplasmin (mg/dL)	41	18-36
Natural killer cell count (cells/μL)	66	70-760
Soluble CD25 (pg/mL)	34,650	531-1,891

**Figure 1:**
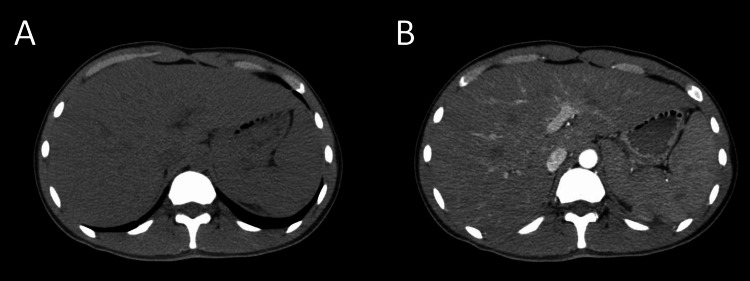
CT scan of the abdomen with IV contrast. (A, B) Axial sequences of the abdomen showing hepatomegaly and splenomegaly before and after IV contrast. CT: computed tomography. IV: intravenous.

An ultrasound of the gallbladder was obtained that showed an acalculous gallbladder with CBD measuring 4 mm. Computed tomography (CT) scan of the chest, abdomen, and pelvis with intravenous contrast revealed hepatosplenomegaly and no lymphadenopathy (Figures 1A, 1B). Further immunological studies including direct antiglobulin test (Coomb’s test), hairy localization element (HLE) gene mutation, anti-smooth muscle antibody, liver-kidney microsomal antibody, antinuclear antibody, anti-mitochondrial antibody, and rheumatoid factor, were obtained and were negative. The transthoracic echocardiogram was unrevealing. Bone marrow biopsy showed normocellular marrow with peripheral pancytopenia without evidence of leukemia or lymphoma. During the course of his hospitalization, the patient developed spikes of moderate- to high-grade fevers and was started on prophylactic antibiotics. Further lab work revealed an EBV viral load of more than 2 million copies/mL as well as a low NK cell count of 66 cells/μL and a high soluble cluster of differentiation 25 (CD25) of 34,650 pg/mL (Table 1). Our patient met the diagnostic criteria for HLH and was started on an induction course of intravenous dexamethasone for eight weeks and was transferred to another facility for a higher level of care for chemotherapy.

## Discussion

We present a young patient who presented with hiccups and abdominal pain who was found to be in a state of an aberrant immune response due to a severe EBV infection. Infections are the most common cause of HLH with EBV being the most common infectious trigger of HLH [2]. Other viruses implicated in HLH include cytomegalovirus, parvovirus, herpes simplex virus, varicella-zoster virus, measles virus, human herpes virus 8, influenza virus, and SARS-CoV-2 [6,14]. Although HLH has been found in association with X-linked lymphoproliferative disease following an EBV infection, the majority of EBV-induced HLH cases occur in previously healthy and immunocompetent children similar to our patient [15].

The classical diagnosis of HLH requires five of the following to be met: fever, splenomegaly, bicytopenia, hypertriglyceridemia and/or hypofibrinogenemia, and hemophagocytosis [16]. In HLH-2004, three additional criteria were introduced: low/absent NK cell activity, hyperferritinemia and high soluble interleukin-2 receptor (IL-2R, also known as CD25) levels [11]. To diagnose HLH, at least five out of the eight criteria must be met. In uncertain cases, the HScore may be utilized to aid the diagnosis [17]. In a cohort of 312 patients, an HScore of 169 corresponded to a sensitivity of 93% and a specificity of 86% and was able to accurately classify 90% of the patients [17]. Our patient fulfilled the criteria for the diagnosis of HLH. Hyperferritinemia is common in HLH as the primary storage site for ferritin is within tissue macrophages [18]. We also noted abnormal liver enzymes in our patient which were previously reported to be present in as high as 98.1% of patients with HLH [19] which is hypothesized to be due to lymphocytic infiltration of the hepatic portal triads [20]. Serum markers have proven to be valuable diagnostic parameters as ferritin levels above 500 µg/L had a sensitivity of 84% and soluble CD25 above 2400 U/mL had a sensitivity of 93% [11]. While we did not observe hemophagocytosis on the bone marrow biopsy, the presence of hemophagocytosis is not required for diagnosis, nor does its absence rule out the disease [21]. Indeed, hemophagocytosis is not considered sensitive or specific [22], is found in approximately 82% of patients with HLH [23] and may be absent in the early stages of the disease [24].

Hypercytokinemia during the natural course of HLH causes a life-threatening hyperinflammatory state [25]. In HLH with underlying genetic etiology, this is attributed to HLH-associated mutations in genes that encode components of the machinery for perforin (PRF)-dependent cytotoxicity [26]. For instance, adult-onset familial HLH was found to be associated with mutations in *PRF1*, mammalian uncoordinated 13-4 (*MUNC13-4*), and syntaxin binding protein 2 (*STXBP2*) [27]. The encoded proteins are involved in the PRF1-dependent cytotoxic pathway, whose mutation results in uncontrolled activation of cytotoxic T lymphocytes and NK cells [28]. These genes modulate the degranulation process following an immune challenge [29]. In the case of a viral stimulus, EBV infection leads to monoclonal or oligoclonal proliferation of B-cells, T-cells, and NK cells [30] with a persistent activation state and a resultant cytokine storm [31]. Since the extrusion of cytotoxic granules against virus-infected cells is impaired in HLH, the intended target cells survive and continue to feed into the initial response leading to a vicious hyperinflammatory cycle and an intensified immune response [32]. The released proinflammatory cytokines and factors such as IL-1β, IL-6, interferon-γ (IFN-γ) and tumor necrosis factor-α (TNF-α) [33], result in macrophage activation, hemophagocytosis, tissue damage, and organ failure [34]. Importantly, higher levels of cytokine expression were found to be associated with poorer outcomes [33], as well as in patients with neurological sequelae [35]. While we did not perform genetic testing on our patient to rule out an underlying genetic predisposition, given his age, the absence of family history of HLH-like disease, in addition to the EBV viral load, we concluded that our patient has HLH caused by an EBV infection.

Early diagnosis and treatment of EBV-induced HLH is imperative as patients may deteriorate rapidly and develop fatal multi-organ failure [36,37]. In a case series of 162 adult patients with HLH, 42% did not survive and half died within one month of diagnosis [10]. According to the HLH 2004 protocol, treatment includes induction therapy with dexamethasone (total of eight weeks: 10 mg/m^2^ for two weeks followed by 5 mg/m^2^ for two weeks then 2.5 mg/m^2^ for two weeks then 1.25 mg/m^2^ for the last two weeks) and renally-dosed etoposide (normally 150 mg/m^2^ per dose: five doses in the first two weeks followed by five doses every week) in addition to intrathecal methotrexate and hydrocortisone in patients with central nervous system (CNS) involvement [1]. Patients with adequate response to the induction course may stop therapy after the eight weeks of treatment, otherwise, a continuation therapy may be considered with or without the addition of cyclosporine. In refractory patients, those with CNS involvement and patients with a known genetic form, hematopoietic cell transplant (HCT) may be pursued [1] and carries a five-year survival rate of 66% in adults with HLH. In our patient who is medically stable, we have elected to pursue a dexamethasone induction course while monitoring response to therapy. Besides plasma exchange and intravenous immunoglobulin, emerging therapies that warrant further clinical investigation include anti-IL-1, anti-TNF-α, anti-IFN-γ treatments [32] as well as kinase inhibitors [32], rituximab [38] and anti-virals [39].

## Conclusions

Our report highlights the tedious diagnostic process of HLH in a previously healthy young patient presenting with non-specific gastrointestinal symptoms. Early diagnosis and treatment of HLH are critical in minimizing the consequences of fulminant HLH with multi-organ failure. Treatment of HLH entails attenuating the pathologic immune response and resultant hypercytokinemia. Further studies are warranted to investigate the role of chemotherapeutic agents and immunosuppressants in EBV-induced HLH.
